# Bleeding and Thrombotic Risk in Low Dose Heparin Infusion as Compared to Standard Dose Heparin Infusion

**DOI:** 10.7759/cureus.8339

**Published:** 2020-05-28

**Authors:** Forat Lutfi, Rohit Bishnoi, Vikas J Patel, Aisha Elfasi, Michael Setteducato, Shuyao Zhang, Chintan P Shah, Saji Kurian, Chethana Kamath, Dae Jun Kim, Marc S Zumberg, Martina Murphy

**Affiliations:** 1 Hematology and Oncology, University of Maryland Medical Center, Baltimore, USA; 2 Hematology and Oncology, University of Florida Health, Gainesville, USA; 3 Gastroenterology, University of Florida Health, Gainesville, USA; 4 Neurology, University of Florida Health, Gainesville, USA; 5 Internal Medicine, University of Florida Health, Gainesville, USA; 6 Internal Medicine, University of Texas Southwestern Medical Center, Dallas, USA

**Keywords:** anticoagulant therapy, heparin, thrombosis, hemorrhage, venous thromboembolism

## Abstract

Intravenous unfractionated heparin (UFH) remains one of the most commonly used anticoagulants in the hospital setting. The optimal protocol for initiation and maintenance of UFH has been difficult to determine. Over the past two decades, weight-based nomogram protocols have gained favor. Herein, we present a retrospective study of 377 patients at a single tertiary academic center treated with low intensity (LI) and standard intensity (SI) UFH protocols for therapeutic anticoagulation. UFH levels are measured by anti-Xa assay activity with therapeutic levels of 0.30 to 0.70 IU/mL for SI and 0.25 to 0.35 IU/mL for LI.

Patients treated on the LI protocol were more likely to have had a previous history of bleeding and lower baseline hemoglobin. Incidence of new or worsening thrombus while on UFH was comparable between both protocols (odds ratio (OR) 0.93, 95% confidence interval (CI) 0.29-2.98, p=0.899). Patients on LI protocol had higher incidence of bleeding while on UFH (OR 1.21, 95% CI 0.51-2.89, p=0.667). Our study thus suggests that the LI protocol may have comparable efficacy to the SI protocol in treating venous thromboembolism (VTE) and that target anti-Xa levels of 0.25 to 0.35 IU/mL may be more optimal in high-risk patients.

## Introduction

Since the discovery of heparin by Howell in 1916 and its initial use on human subjects in 1935, it has been one of the most commonly utilized inpatient medications in modern medicine [1,2]. However, despite its frequent inpatient use and importance in preventing and treating venous thromboembolism (VTE), therapeutic protocols for unfractionated heparin (UFH) have varied between institutions and organizations. Given the importance of the use of therapeutic heparin in treating VTE and minimizing the risk of hemorrhage posed by its use, determining the optimal protocol is of the utmost importance.

Determining the optimal protocol for UFH bolus and subsequent infusion has been controversial. In 1989, the American College of Chest Physicians (ACCP) Clinic Practice Guideline on VTE treatment recommended an initial bolus of 5,000-units followed by 1000-units/hour [3]. More recently, guidelines have incorporated weight-based recommendations [4-12]. The 2012 ACCP guidelines published as the 9th edition in Chest recommend an initial bolus of 80-units/kg followed by 18 units/kg/hr adjusted to therapeutic levels [13]. 

In patients deemed high-risk for hemorrhage, a low intensity (LI) rather than standard intensity (SI) protocol is often employed. However, to date, there has been little study of differences in adverse events, namely hemorrhage, and efficacy between LI and SI protocols. Furthermore, identifying the effect of patient-specific factors (e.g. age, indication for UFH, anticoagulant and anti-platelet use, medical, and surgical history) on outcomes has the potential to assist in determining the most appropriate protocol.

## Materials and methods

A total of 377 adult patients receiving therapeutic UFH from July 2011 to July 2017 at a single tertiary academic center were retrospectively studied. Patients receiving UFH on a separate acute coronary syndrome protocol and those receiving concomitant thrombolytic agents were excluded. Only those patients treated on LI or SI UFH protocols were included in analysis. Indications for LI or SI UFH use was VTE (deep venous thrombosis (DVT) or pulmonary embolism (PE)), atrial fibrillation, acute coronary syndrome, arterial thrombus, cerebral ischemic event, portal vein thrombosis, and for cardiac valves.

At our institution, UFH is administered by SI (therapeutic target anti-Xa activity level 0.30 to 0.70 IU/mL) and LI (therapeutic target anti-Xa activity level 0.25 to 0.35 IU/mL) protocols. The anti-Xa activity assay is used preferentially over activated partial thromboplastin time (aPTT) as a method of determining in vivo heparin activity [14-19]. Both protocols are initiated with a weight-based bolus, followed by an initial infusion rate of 12 or 18 units/kg/hr for LI and SI protocols, respectively. UFH is dosed based on actual body weight if the patient weight is less than 125kg. For patients weighting greater than 125 kg, adjusted body weight is used to determine dosing (Table 1).

**Table 1:** Nursing protocol for therapeutic infusion of unfractionated heparin (UFH) Note: heparin unfractionated levels are obtained six hours from initiation of the infusion and then checked every six hours until therapeutic on two consecutive measurements. Thereafter, heparin unfractionated levels are obtained every morning. *Total body weight is used for dose calculation if ≤125 kg, if >125 kg, adjusted body weight is used to determine dosing.

Low Intensity:	Standard Intensity:
Optional bolus: 60 units/kg	Suggested bolus: 80 units/kg
Initial infusion rate: 12 units/kg/hr	Initial infusion rate: 18 units/kg/hr
UFH <0.11: bolus of 60 units/kg units and increase rate by 3 units/kg/hr	UFH <0.2: bolus of 80 units/kg and increase rate by 4 units/kg/hr
UFH 0.11-0.24: bolus of 30 units/kg and increase rate by 2 units/kg/hr	UFH 0.2-0.29: bolus of 40 units/kg and increase rate by 2 units/kg/hr
UFH 0.11-0.24: bolus of 30 units/kg and increase rate by 2 units/kg/hr	UFH 0.3-0.7: at goal, no changes
UFH 0.25-0.35: at goal, no change	UFH 0.71-0.8: decrease rate by 1 units/kg/hr
UFH 0.36 to 0.55: decrease rate by 2 units/kg/hr	UFH 0.81-0.9: stop for 30 min and decrease by 2 units/kg/hr
UFH >0.56: stop infusion for 60 min and decrease rate by 3 units/kg/hr	UFH >0.91: stop infusion for 60 min and decrease by 3 units/kg/hr

Intravenous infusion of UFH is ordered using an Electronic Medical Record (EMR) order set involving all steps for the monitoring of heparin concentrations measured in units/mL using an anti -Xa assay. The decision on whether to use LI or SI protocols is based on the ordering physician’s discretion and their personal assessment of the patient’s bleeding and thrombotic risk and treatment indication.

Chart review was done manually using the EMR and data was recorded in REDCap to standardize data collection and decrease researcher variability. REDCap is a secure web application for building and managing online databases. The primary outcomes measured were incidence of new or worsening thrombus and/or bleeding while receiving intravenous UFH. Bleeding was categorized into major and minor, based on the International Society on Thrombosis and Haemostasis (ISTH) definitions [20]. Secondary outcomes measured include the incidence of transfusion requirement within one month and the incidence of death within three months of initiation of UFH. IRB approval was obtained prior to collection of data (University of Florida IRB201702116). Data analysis was conducted using the SAS 9.4 statistical software (SAS Institute Inc., Cary, North Carolina). Descriptive statistics were determined for each study variable. Univariate analysis was applied to identify the risk factors associated with the outcome variable. The Fisher exact test was used to compare the geographic categorical variables between the outcome groups and the independent t-test was used to compare the numerical variables between the outcome groups. If the underlying assumptions of the t-test were violated, the corresponding nonparametric test (Wilcoxon rank-sum test) was used to replace the t-test. The statistically significant (p< .05) variables in the univariate analysis phase were selected to build a multivariable logistic regression model. The stepwise selection procedure was used for the model selection.

## Results

Of the 377 patients studied, 42.0% (158) and 58.0% (219) were on LI and SI protocols, respectively. The majority of patients (76.1%) received an initial bolus of 60 units/kg (LI) or 80 units/kg (SI) with a higher prevalence of bolus in the SI group (84.0% versus 65.2%.) Median time to therapeutic levels was 5.5 hours in the LI group and 3.3 hours in the SI group (Table 2).

Patients were predominately Caucasian (74.0%) with median age of 63 (range of 19-93) years-old. Gender and body mass index (BMI) were similar in both groups. The main indications for therapeutic UFH were VTE (46.9%) and atrial fibrillation (18.6%). The indication for UFH was comparable between both groups with the exception of a higher percentage of those on SI protocol being treated for VTE (53.4% versus 38.0%) (Table 2).

Many patients were on home anti-platelet (35.0%) and anticoagulant (33.2%) therapy prior to admission. The percentage of patients on aspirin, anti-platelet, and injectable anticoagulants was similar in both groups. A higher percentage of patients on LI protocol were on oral anticoagulants (36.1% versus 24.2%). LI protocol patients were more likely to have had a history of previous bleeding (24.1% versus 12.8%). HAS-BLED scores (hypertension, abnormal renal/liver function, stroke, bleeding history or predisposition, labile international normalized ratio, elderly, drugs/alcohol concomitantly) were comparable with a median score of 2 and range of 0-6 in LI and 0-7 in SI groups, respectively. Documented history of peptic ulcer disease within three months of heparin initiation was comparable in both groups (Table 2).

Initial hemoglobin and platelet count was lower in the LI group. Median international normalized ratio (INR) was the same in both groups with a slightly greater range in the SI group. Initial median and range PTT was similar in both groups. Patients with active malignancy were comparable in both groups (24.1% versus 21.0%). A significantly higher percentage of patients on LI protocol had a recent surgery (34.8% versus 11.9%). There was a higher percentage of active smokers in the SI group (20.5% versus 11.4%) (Table 2).

**Table 2:** Baseline patient characteristics BMI: body mass index; UFH: unfractionated heparin; VTE: venous thromboembolism; NSAIDs: nonsteroidal anti-inflammatory drugs; PPT: partial thromboplastin time; INR: international normalized ratio; HAS-BLED scores: hypertension, abnormal renal/liver function, stroke, bleeding history or predisposition, labile international normalized ratio, elderly, drugs/alcohol concomitantly.

Variable:	Low Intensity: (N=158)	Standard Intensity: (N=219)
Age median (range):	63.5 (19.0-92.0) years-old	63.0 (23.0-93.0) years-old
Gender :		
Male:	83 (52.5%)	119 (54.3%)
Female:	75 (47.5%)	100 (45.7%)
Race:		
Caucasian:	123 (77.9%)	156 (71.2%)
African American:	28 (17.7%)	53 (24.2%)
Other:	7 (4.4%)	10 (4.6%)
BMI:	28.0 (16.6-53.0) kg/m^2^	28.0 (15.8-55.5) kg/m^2^
Initial bolus of UFH:	103 (65.2%)	184 (84.0%)
Time to reach therapeutic level median (range):	5.5 (0.1-138.6) hours	3.3 (0.3-20.8) hours
Indication for UFH:		
VTE:	60 (38.0%)	117 (53.4%)
Atrial fibrillation:	30 (19.0%)	40 (18.3%)
Arterial thrombus:	14 (8.9%)	10 (4.6%)
Heart valve:	15 (9.5%)	8 (3.7%)
Ischemic stroke:	16 (10.1%)	11 (5.0%)
Acute coronary syndrome:	10 (6.3%)	16 (7.3%)
Intra-cardiac thrombus:	8 (5.1%)	8 (3.7%)
Portal vein thrombus:	5 (3.2%)	9 (4.1%)
Home medications:		
Aspirin:	45 (28.5%)	65 (29.7%)
Anti-platelet agent:	53 (33.5%)	79 (36.1%)
Oral anticoagulants:	57 (36.1%)	53 (24.2%)
Injectable anticoagulants:	7 (4.4%)	8 (3.7%)
NSAIDs:	7 (4.4%)	16 (7.3%)
Previous history of bleeding:	38 (24.1%)	28 (12.8%)
HAS-BLED score median (range):	2 (0-6)	2 (0-7)
Peptic Ulcer Disease:	6 (3.8%)	7 (3.2%)
Initial Hemoglobin/Hematocrit (range):	9.8g/dL/29.6% (5.9-17.3g/dL/17.4-50.8%)	11.6g/dL/35.0% (4.9-17.0g/dL/15.3-49.4%)
Initial platelet count (range):	192.5g/cm^3^ (48.0-982.0g/cm^3^)	202.0g/cm^3^ (46.0-971.0g/cm^3^)
Initial PTT median (range):	33.0 (21.0-240.0)	32.0 (13.5-240.0)
Initial INR median (range):	1.2 (0.9-2.5)	1.2 (0.9-5.4)
Active malignancy:	38 (24.1%)	46 (21.0%)
Surgery within 1-week of starting UFH:	55 (34.8%)	26 (11.9%)
Current smoker:	18 (11.4%)	45 (20.5%)

The incidence of new or worsening thrombosis was comparable between both protocols (3.2% versus 3.7%). Incidence of bleeding was 44% higher in the LI group (11.4% versus 7.3%). On average, the last anti-Xa level prior to bleeding was therapeutic per both intensity protocols (0.30 versus 0.60). The LI group had 58% higher transfusion rates within one month (29.7% versus 16.4%) (Table 3).

**Table 3:** Bleeding and thrombosis while on therapeutic heparin UFH: unfractionated heparin.

Variable:	Low Intensity N (%, 95% CI):	Standard Intensity N (%, 95% CI):
New or worsening thrombus while on UFH:	5 (3.2%, 1.0-7.2%)	8 (3.7%, 1.6-7.1%)
Bleeding while on UFH:	18 (11.4%, 6.9-17.4%)	16 (7.3%, 4.2-11.6%)
Last anti-Xa level prior to bleed (range):	.30 (0.0-.60)	.60 (0.0-1.1)
Transfusion requirement within one-month:	47 (29.7%)	36 (16.4%)
Death in three-months:	25 (15.8%)	24 (11.0%)

New bleeding events were more likely to occur than recurrent bleeding. The incidence of major bleeding was more likely in LI compared with SI (5.0% versus 2.7%). The most frequent site of bleeding was gastrointestinal for both protocols (Table 4).

**Table 4:** Bleeding while on unfractionated heparin (UFH) *Major bleeding defined as bleeding that is fatal, involves a critical organ (intraspinal, intracranial, retroperitoneal, or pericardial) or that causes a >2 g/dL decline in hemoglobin or requires transfusion of red blood cells.

Variable:	Low Intensity:	Standard Intensity:
Chronicity:		
New:	16 (10.1%)	14 (6.4%)
Recurrent:	2 (1.2%)	2 (0.9%)
*Grade:		
Major:	8 (5.0%)	6 (2.7%)
Minor:	10 (6.3%)	10 (4.6%)
Bleed location:		
Gastrointestinal:	5 (3.2%)	3 (1.4%)
Genitourinary:	2 (1.2%)	2 (0.9%)
Surgical Site:	5 (3.2%)	2 (0.9%)
Retroperitoneal:	1 (0.6%)	2 (0.9%)
Epistaxis:	2(1.2%)	0 (0%)
Soft tissue/musculoskeletal:	1 (0.6%)	2 (0.9%)
Other:	2 (1.2%)	5 (2.3%)

The odds of experiencing new or worsening thrombus while on UFH was comparable (odds ratio (OR) 0.93, 95% confidence interval (CI) 0.29-2.98, p=0.899) in both groups. In multivariate analysis, the odds of bleeding while on LI was 1.21 times (95% CI 0.51-2.89) more likely than SI (p=0.667). Mortality at three-months from initiation of UFH was also comparable (OR 0.95, 95% CI 9.50-1.80, p=0.872) (Table 5).

**Table 5:** Multivariate analysis of clinical outcomes, low intensity (LI) vs standard intensity (SI) UFH: unfractionated heparin.

Variable:	Odds Ratio (95% CI):	p-value:
New or worsening thrombus while on UFH:	0.93 (0.29-2.98)	0.899
Bleeding while on UFH:	1.21 (0.51-2.89)	0.667
Death in three-months:	0.95 (0.50-1.80)	0.872

## Discussion

The use of UFH remains an important modality in treatment and prophylaxis of VTE. Throughout the years, determining the optimal treatment protocol, therapeutic levels, and assays of measurement has been difficult to ascertain. To date, there does not exist a single, monolithic protocol, universally implemented at all institutions. However, guidelines with both weight-based and non-weight-based nomograms have been proposed [4-12]. The more recent 2012 ACCP guidelines follow a weight-based nomogram and are classified as a Grade 2C recommendation [13]. They do not, however, have a recommended therapeutic level range or recommended assay of measurement in adult patients. In neonates and children, there is a Grade 2C recommendation of an anti-Xa activity level of 0.35-0.70 units/mL [21]. Furthermore, a scoring system to risk-stratify patients for bleeding on UFH, similar to the HAS-BLED score for atrial fibrillation, does not exist.

At the University of Florida (Gainesville, FL), a tertiary academic center, intravenous UFH is administered by SI and LI protocols with target anti-Xa activity levels of 0.30 to 0.70 IU/mL and 0.25 to 0.35 IU/mL, respectively. These protocols include a suggested bolus, standard initial infusion rates, and titration parameters until a therapeutic range is reached as noted in Table 1. The development of this protocol occurred in close collaboration with Hematology faculty and staff pharmacy based on previously published weight-based UFH nomograms and clinical practice recommendations [19,22-27].

Given limited available data on the efficacy and risks of the implemented protocol at our institution and others, investigators designed a review of 377 patients treated with intravenous UFH. The primary endpoints studied were new or worsening thrombosis and bleeding. The incidence of new or worsening thrombus was low and similar in both groups and not statistically significant (3.2% versus 3.7%, OR 0.93, p=0.899). The incidence of bleeding was higher in the LI group (11.4% versus 7.3%) with those on LI being 1.21 times more likely to experience bleed than the SI group, although not statistically significant (p=0.667). The LI group was more likely to have had a previous history of bleed and had lower average baseline hemoglobin/hematocrit (9.8 g/dL/29.6% versus 11.6 g/dL/35.0%). The LI group was more likely to require transfusion within one month (29.7% versus 16.4%). The increased incidence of bleeding and transfusion requirements in the LI group was likely due to the higher-risk population selected for a low intensity protocol.

Given that the decision to use a protocol was based solely on clinician judgement, there was a desire to see if an existing scoring system could be utilized to better serve clinicians in deciding the most appropriate protocol. The existing HAS-BLED scoring system was thus used. The HAS-BLED system was developed to assess the one-year risk of major bleeding events in patients on anticoagulation for atrial fibrillation [28]. Interestingly, the HAS-BLED median score and range were nearly identical in both LI and SI groups despite notable differences in bleeding history and incidence. This suggests that the HAS-BLED scoring system is not applicable in determining the risk of bleeding in patients on UFH. The necessity of developing an applicable scoring system for patients on UFH for VTE remains.

We acknowledge multiple limitations in this project, including its retrospective nature without randomization. However, many baseline patient characteristics (e.g. age, gender, BMI, indication for anticoagulation) were comparable between both groups. The LI population was higher risk for bleeding based on previous history and comorbidities and thus explains the physician’s choice of LI protocol and the higher noted incidence of bleeding while on UFH. This higher risk of bleeding in patients receiving the LI protocol also likely explains the increased incidence of bleeding when compared to the SI protocol.

## Conclusions

In conclusion, a LI protocol may have comparable efficacy to a SI protocol based on the similar incidence of new or worsening thrombus. This is of notable importance in patients at high risk for bleeding. In the future, a prospective randomized trial of both protocols matched for comorbidities, risk factors, and indications is needed to further elaborate on the findings of this study with the ultimate goal of developing a risk stratification scoring system and weight-based dosing nomogram for use by clinicians. 

## References

[1] Li JJ, Corey EJ (2013). Drug Discovery: Practices, Processes, and Perspectives. https://www.wiley.com/en-us/Drug+Discovery%3A+Practices%2C+Processes%2C+and+Perspectives-p-9780470942352.

[2] (2020). WHO Model Lists of Essential Medicines. https://www.who.int/medicines/publications/essentialmedicines/en/.

[3] Hyers TM, Hull RD, Weg JG (1989). Antithrombotic therapy for venous thromboembolic disease. Chest.

[4] Raschke RA, Reilly BM, Guidry JR, Fontana JR, Srinivas S (1993). The weight-based heparin dosing nomogram compared with a “standard care” nomogram: a randomized controlled trial. Ann Intern Med.

[5] Shalansky KF, FitzGerald JM, Sunderji R, Traboulay SJ, O’Malley B, McCarron BI, Naiman S (1996). Comparison of a weight-based heparin nomogram with traditional heparin dosing to achieve therapeutic anticoagulation. Pharmacotherapy.

[6] Schlicht JR, Sunyecz L, Weber RJ, Tabas GH, Smith RE (1997). Reevaluation of a weight-based heparin dosing nomogram: is institution-specific modification necessary?. Ann Pharmacother.

[7] Lackie CL, Luzier AB, Donovan JA, Feras HI, Forrest A (1998). Weight-based heparin dosing: clinical response and resource utilization. Clin Ther.

[8] Büller HR, Agnelli G, Hull RD, Hyers TM, Prins MH, Raskob GE (2004). Antithrombotic therapy for venous thromboembolic disease. Chest.

[9] Kearon C, Kahn SR, Agnelli G, Goldhaber S, Raskob GE, Comerota AJ (2008). Antithrombotic therapy for venous thromboembolic disease. Chest.

[10] Geerts WH, Pineo GF, Heit JA, Bergquist D, Lassen MR, Colwell CW, Ray JG (2004). Prevention of venous thromboembolism. Chest.

[11] Price CK, Colodny L (2000). Partnering with nurses to manage heparin therapy with a weight-based protocol. Am J Health Syst Pharm.

[12] Sherman DS, Clarke SH, Lefkowitz JB, Valuck RJ, Lindenfeld JA, Stringer KA (2001). An institution-specific heparin titration nomogram: development, validation, and assessment of compliance. Pharmacotherapy.

[13] Guyatt GH, Akl EA, Crowther M, Schünemann HJ, Gutterman DD, Lewis SZ (2012). Introduction to the ninth edition: antithrombotic therapy and prevention of thrombosis, 9th ed: American College of Chest Physicians evidence-based clinical practice guidelines. Chest.

[14] Francis JL, Groce JB III (2004). Challenges in variation and responsiveness of unfractionated heparin. Pharmacotherapy.

[15] Lehman CM, Frank EL (2009). Laboratory monitoring of heparin therapy: partial thromboplastin time or anti-Xa assay?. Lab Med.

[16] Warkentin TE (2014). Anticoagulant failure in coagulopathic patients: PTT confounding and other pitfalls. Expert Opin Drug Saf.

[17] Levine MN, Hirsh J, Gent M (1994). A randomized trial comparing activated thromboplastin time with heparin assay in patients with acute venous thromboembolism requiring large daily doses of heparin. Arch Intern Med.

[18] Rosborough TK (1999). Monitoring unfractionated heparin therapy with antifactor Xa activity results in fewer monitoring tests and dosage changes than monitoring with the activated partial thromboplastin time. Pharmacotherapy.

[19] Guervil DJ, Rosenberg AF, Winterstein AG, Harris NS, Johns TE, Zumberg MS (2011). Activated partial thromboplastin time versus antifactor Xa heparin assay in monitoring unfractionated heparin by continuous intravenous infusion. Ann Pharmacother.

[20] Kaatz S, Ahmad D, Spyropoulos AC, Schulman S (2015). Definition of clinically relevant non-major bleeding in studies of anticoagulants in atrial fibrillation and venous thromboembolic disease in non-surgical patients: communication from the SSC of the ISTH. J Thromb Haemost.

[21] Guyatt GH, Akl EA, Crowther M, Gutterman DD, Schuünemann HJ (2012). Antithrombotic therapy and prevention of thrombosis, 9th ed: American College of Chest Physicians Evidence-Based Clinical Practice Guidelines. Chest.

[22] Hull RD, Raskob GE, Hirsh J, Jay RM, Leclerc JR, Geerts WH (1986). Continuous intravenous heparin compared with intermittent subcutaneous heparin in the initial treatment of proximal-vein thrombosis. N Engl J Med.

[23] Rosenberg AF, Zumberg M, Taylor L, Leclaire A, Harris N (2010). The use of anti-xa assay to monitor intravenous unfractionated heparin therapy. J Pharm Pract.

[24] Brill-Edwards P, Ginsberg JS, Johnston M, Hirsh J (1993). Establishing a therapeutic range for heparin therapy. Ann Intern Med.

[25] Smith ML, Wheeler KE (2010). Weight-based heparin protocol using antifactor Xa monitoring. Am J Heal Pharm.

[26] Tahir R (2007). A review of unfractionated heparin and its monitoring. US Pharm.

[27] Cruickshank MK, Levine MN, Hirsh J, Roberts R, Siguenza M (1991). A standard heparin nomogram for the management of heparin therapy. Arch Intern Med.

[28] Pisters R, Lane DA, Nieuwlaat R, de Vos CB, Crijns HJGM, Lip GYH (2010). A novel user-friendly score (HAS-BLED) to assess 1-year risk of major bleeding in patients with atrial fibrillation. Chest.

